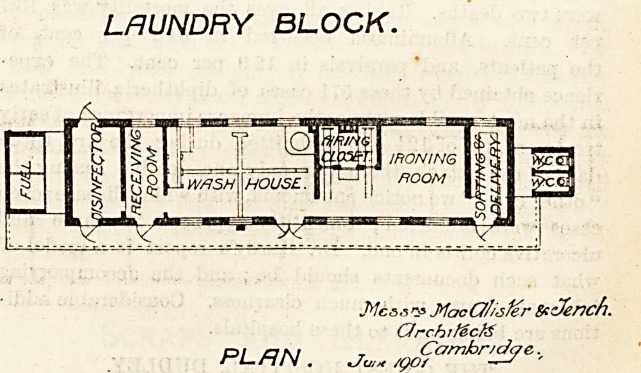# The Sanatorium, Cambridge

**Published:** 1901-09-28

**Authors:** 


### THE INSTITUTIONAL WORKSHOP.

### THE SANATORIUM, CAMBRIDGE.

ENLARGEMENTS are now in course of erection at the isolation hospital in Cambridge. These comprise a nurses' home, a laundry, and a block for the treatment and isolation of diphtheria patients. The latter is a well-designed building consisting of a centre and two wings. In the centre are the hall, bath-room, clothes store, and nurses' duty room. From the latter projects the operating-room. This is octagonal in shape, and there are windows in four of its sides ensuring plenty of light. Each wing consists of a ward for four beds, and a sanitary block containing closet, lavatory, and sink. These latter blocks are correctly cut off from the wards by ventilating passages. Each of the wards has three windows on two of its sides. These windows being placed directly opposite to each other permit of proper cross-ventilation of the ward, and show that the correct principle of having windows on both sides of every bed has been carried out—a provision not too common in small hospitals. The heating is by "warm air ventilating stoves." Presumably these have open fires—at least, we hope so. The floors are of papyrolith throughout, and the walls are of plaster varnished. Why not cement?

The nurses' home contains accommodation for matron, twelve nurses, and six domestics. It also, with the exception of the upstairs sanitary arrangements, is a well-designed block. The same may be said of the laundry. Indeed, there is a directness and simplicity about these plans which we look on with pleasure.

The architects are Messrs. MacAlister and Tench, of Cambridge. The cost of the three blocks is £7,000.

GROUND FLOOR PLAN. FIRST FLOOR PLAN.

## Figures and Tables

**Figure f1:**
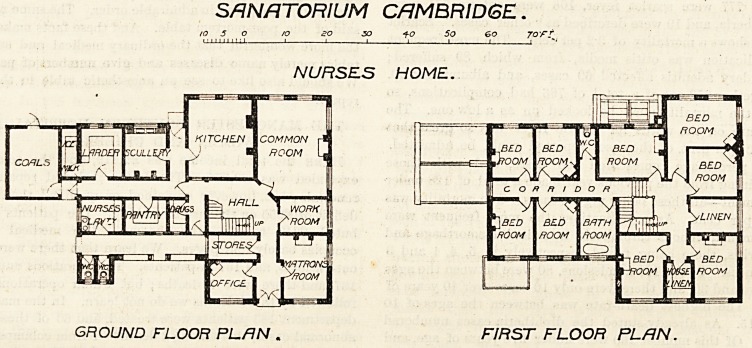


**Figure f2:**
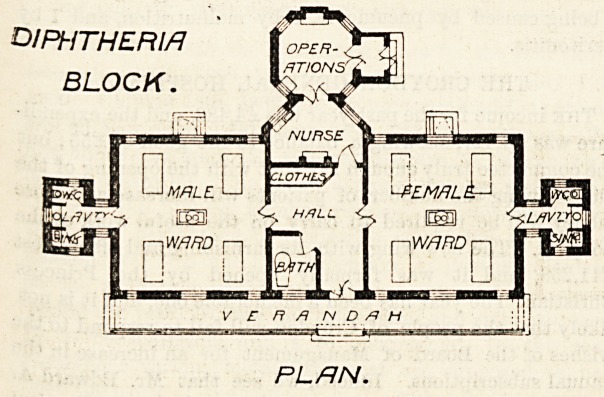


**Figure f3:**